# Revisiting Hepatic Fibrosis Risk in Congenital Heart Disease: Insights from Non-Invasive Markers and Echocardiography

**DOI:** 10.3390/children12091131

**Published:** 2025-08-27

**Authors:** Fusako Yamazaki, Hiroteru Kamimura, Saori Endo, Suguru Miida, Hiroki Maruyama, Tomoaki Yoshida, Masaru Kumagai, Naruhiro Kimura, Hiroyuki Abe, Akira Sakamaki, Takeshi Yokoo, Masanori Tsukada, Fujito Numano, Akihiko Saitoh, Maya Watanabe, Shuichi Shiraishi, Masanori Tsuchida, Shinya Fujiki, Takeshi Kashimura, Takayuki Inomata, Hirofumi Nonaka, Kenya Kamimura, Atsunori Tsuchiya, Shuji Terai

**Affiliations:** 1 Division of Gastroenterology and Hepatology, Graduate School of Medical and Dental Sciences, Niigata University, Niigata 951-8510, Japan; fu.r.j.n.7240@gmail.com (F.Y.); saka-a@med.niigata-u.ac.jp (A.S.);; 2 Department of Pediatrics, Graduate School of Medical and Dental Sciences, Niigata University, Niigata 950-3198, Japan; 3 Division of Thoracic and Cardiovascular Surgery, Graduate School of Medical and Dental Sciences, Niigata University, Niigata 950-3198, Japan; 4 Department of Cardiovascular Medicine, Niigata University Medical and Dental Hospital, Niigata 950-3198, Japan; 5 Department of Business Administration, Aichi Institute of Technology, Toyota 470-0356, Japan; 6 Department of General Medicine, School of Medicine, Niigata University, Niigata 950-3198, Japan; kenya-k@med.niigata-u.ac.jp; 7 Department of Gastroenterology and Hepatology, Faculty of Medicine, University of Yamanashi, Kofu 400-0016, Japan

**Keywords:** aspartate-to-platelet ratio index, congenital heart disease, Fontan circulation, hepatic fibrosis markers, tetralogy of Fallot

## Abstract

**Highlights:**

**What are the main findings?**

- The prevalence of liver fibrosis increased stepwise across CHD subtypes, with APRI > 0.5 observed in 12.7% of VSD, 43.3% of TOF, and 67.9% of Fontan patients.
- Non-invasive markers including γ-GTP ≥ 53 U/L and BNP ≥ 35.5 pg/mL showed high specificity in identifying patients at risk of liver fibrosis.

**What is the implication of the main finding?**

- APRI, γ-GTP, and BNP, combined with echocardiographic findings such as absence of IVC respiratory variation, may serve as a practical triad for early detection of subclinical liver fibrosis in CHD survivors.
- These simple, routinely available indicators can facilitate timely hepatology referral and interdisciplinary care planning across pediatric and adult services.

**Abstract:**

Background/Objectives: This study aimed to investigate the prevalence of liver damage and its associated non-invasive markers and echocardiographic risk factors in patients who underwent surgery for congenital heart disease. Methods: This retrospective observational study was conducted at a single tertiary-care university hospital in Niigata, Japan. Of 142 patients (ventricular septal defect [VSD] n = 47, tetralogy of Fallot [TOF] n = 67, Fontan n = 28), 52.8% were male [median age: 22.7 years; VSD (24.3 years), TOF (24.0 years), and Fontan (12.5 years)]. Pediatric patients with liver diseases unrelated to congestive liver disease, such as viral hepatitis and alcoholic liver disease, were excluded. We compared non-invasive liver fibrosis age-invariant biomarkers, such as the aspartate aminotransferase-to-platelet ratio index (APRI), and various serum markers and echocardiographic parameters to assess the prevalence and predictors of hepatic fibrosis. Results: The Fontan circulation group had the highest APRI, followed by the TOF group, while the VSD group had a low risk of APRI elevation. Postoperative TOF patients required monitoring for cirrhosis progression. Inferior vena cava mobility was associated with echocardiographic parameters and fibrosis severity, along with a loss of respiratory variability. The limitations of other cardiac assessments were highlighted by poor anatomical measurements. Gamma-glutamyl transpeptidase (γ-GTP) demonstrated strong discriminatory ability. The optimal cutoff value was 53.0 U/L, suggesting its use as a clinical marker. Conclusions: Assessing fibrosis is crucial in CHD patients, especially those with late post-TOF repair findings. Non-invasive markers (APRI, γ-GTP, and B-type natriuretic peptide), along with echocardiographic findings, may help detect fibrosis early, enabling timely intervention and improving long-term outcomes. Clinical trial registration: 2020-0199.

## 1. Introduction

Advances in CHD therapy have greatly improved pediatric survival, allowing more patients to reach adulthood. However, adult CHD survivors remain at risk for arrhythmia, infective endocarditis, pregnancy-related complications, and hemodynamic fluctuations that may lead to liver disease. They require lifelong, individualized management tailored to their childhood treatments. To optimize care and outcomes, it is essential to establish dedicated adult CHD centers, strengthen professional education and research, and widely disseminate expertise in adult CHD [1].

Globally, ventricular septal defects (VSDs) are the most frequent CHD subtype, comprising approximately 35.6% of cases (3.07 per 1000 live births), followed by atrial septal defect (ASD, ~15.4%), patent ductus arteriosus (PDA, ~10.2%), pulmonary stenosis (PS, ~6.2%), and tetralogy of Fallot (TOF, ~4.4%) [2].

**Figure 1:**
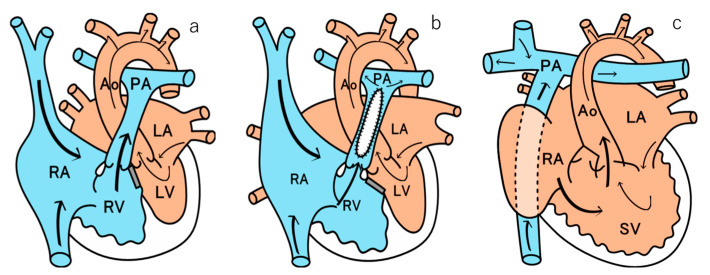
Schematic diagrams of the heart after (**a**) VSD surgery, (**b**) TOF surgery, and (**c**) Fontan surgery. VSD, ventricular septal defect; TOF, tetralogy of Fallot; RA, right atrium; RV, right ventricle; LA, left atrium; LV, left ventricle; PA, pulmonary artery; AO, aorta. Blue indicates venous blood, while red indicates arterial blood.

CHD can be broadly classified into “acyanotic” and “cyanotic” heart disease. Acyanotic heart disease accounts for 60–70% of all CHDs and includes conditions such as atrial septal defect, VSD (Figure 1a), atrioventricular septal defects, and patent ductus arteriosus. Cyanotic heart disease includes conditions such as TOF (Figure 1b), transposition of the great arteries, tricuspid atresia, and pulmonary atresia, which are typically detected in early childhood [3].

Fontan surgery is performed in cyanotic CHD patients with functional single-ventricle hemodynamics, such as a single ventricle, pure pulmonary valve obstruction, tricuspid regurgitation (TR), or hypoplastic left heart syndrome. This procedure involves redirecting blood from the vena cava to the hepatic vein and, subsequently, into the right pulmonary artery (Figure 1c) [4].

A previous study reported a case of cirrhosis during autopsy of a patient who died from fatal arrhythmia 21 months after undergoing Fontan surgery [5]. The hemodynamic impact of CHD surgeries on the liver, including Fontan-associated liver disease (FALD), remains unclear. However, with more cases of concurrent hepatocellular carcinoma in these patients being reported, the underlying mechanisms are getting clarified [6,7].

The European Association for the Study of the Liver has developed guidelines for FALD, and its underlying mechanisms are being increasingly explored [8]. These guidelines list five mechanisms in FALD as follows: hepatic congestion [9], hypoxia and hepatic ischemia [10], accelerated thrombogenic states [11], lymphatic congestion [12], and systemic inflammation [13]. Chronic passive congestion and cardiac cirrhosis worsen with time after the Fontan procedure. Earlier, all examined patients showed evidence of cardiac cirrhosis 4–18 years post surgery [14]. In Fontan circulation, blood flows directly from the veins to the lungs, bypassing the right ventricle. Consequently, blood flow pressure cannot be reliably measured via catheterization, making liver fibrosis assessment using standard echocardiography challenging [15].

TOF is the most common form of cyanotic CHD. Surgical intervention, specifically intracardiac repair, alleviates blood flow restriction and improves circulation from the right ventricle to the lungs. This hemodynamic improvement helps mitigate symptoms such as cyanosis and dyspnea, ultimately enhancing the long-term prognosis of TOF patients [16,17]. However, postoperative complications, including pulmonary artery stenosis and pulmonary valve regurgitation, may occur, leading to an increased right heart load. Gradually, this can progress to right heart failure, mimicking the elevated central venous pressure (CVP) observed after the Fontan procedure (Figure 1b) [18].

Although less frequently reported than FALD, several cases of hepatocellular carcinoma have also been documented following TOF repair [19].

Unlike the single-ventricle anatomy associated with the Fontan procedure, TOF allows measurement of multiple parameters using echocardiography. With an increase in the number of adults with CHD, including those undergoing post-Fontan procedure or TOF repair, establishing standardized assessments for liver fibrosis is crucial. Therefore, identifying simple diagnostic markers usable in daily clinical practice is essential.

Currently, non-invasive assessments of liver fibrosis in pediatric patients remain underdeveloped, making it challenging to determine the optimal timing for hepatology consultations. Therefore, in this study, we aimed to assess the prevalence of liver fibrosis and its associated non-invasive markers and echocardiography in patients who underwent CHD surgery such as VSD, TOF, and Fontan circulation.

## 2. Materials and Methods

### 2.1. Study Design and Patients

This retrospective, observational study was conducted at Niigata University Medical and Dental Hospital between 1 January 1997 and 31 December 2020. It focused on three patient groups: those who underwent VSD repair, TOF repair, and Fontan surgery. Pediatric patients with liver diseases unrelated to congestive liver disease, such as viral hepatitis and alcoholic liver disease, were excluded.

Differences in patient backgrounds, blood test results, fibrosis markers, and cardiac function test results were analyzed across various conditions and study groups.

We performed a matched retrospective cohort analysis wherein patients with VSD—the most common congenital heart malformation—served as a comparison group. VSD cases were matched 1:1 to our primary cohort based on postoperative interval and sex, and all had achieved near-normal cardiac anatomy following surgical repair. Clinical and anatomical parameters were then compared between groups, incorporating hematologic and biochemical profiles, chest radiography, and echocardiography. Noninvasive laboratory indices, including standard liver function tests, were analyzed. Early hepatic fibrosis was quantified using the aspartate aminotransferase-to-platelet ratio index (APRI).

For post-TOF patients, comparative analyses were performed between those with a single-ventricle anatomy who underwent multiple surgeries, including the Fontan procedure, and those with other structural conditions. Factors associated with elevated APRI were evaluated based on the availability of standardized echocardiographic data. Background analysis, including echocardiographic parameters, was performed to identify factors distinguishing the normal-APRI group from the elevated-APRI group.

### 2.2. Chest Radiography

The cardiothoracic ratio (CTR) was measured using posteroanterior chest radiographs taken in full inspiration within 1 month before and after blood sample collection.

### 2.3. Echocardiography

Cardiac function was assessed using parameters reflecting both systolic and diastolic function as well as structural measurements. This approach aligns with the recommendations for cardiac cavity measurements developed by a joint group of the American Society of Echocardiography and European Society of Cardiovascular Imaging [20]. Left ventricular systolic function was evaluated using ejection fraction, which quantifies the percentage of blood ejected from the left ventricle during systole. Diastolic function was evaluated by calculating the E/A ratio (ratio of early to late mitral inflow velocities) and the E/e’ ratio (ratio of early mitral inflow velocity to early diastolic velocity of the mitral annulus), both of which are indicators of left ventricular filling pressure.

Left ventricular dimensions were measured using the internal diameter of the left ventricle at end-diastole and end-systole. Additionally, interventricular septal thickness and posterior wall thickness were measured to assess myocardial hypertrophy. The left ventricular mass index was calculated by normalizing left ventricular mass to body surface area.

Right ventricular function was assessed by measuring the right ventricular internal diameter and evaluating right ventricular systolic function using the tricuspid annular plane systolic excursion (TAPSE). Right atrial diameter was also measured to assess right atrial enlargement.

Left atrial function and size were evaluated using the left atrial diameter and left atrial volume index. The tricuspid regurgitant pressure gradient was measured to assess TR severity and its impact on right ventricular pressure. The presence and severity of valvular regurgitation—including mitral regurgitation, aortic regurgitation, aortic stenosis, and pulmonary regurgitation—were quantified using standard echocardiographic techniques.

Respiratory variation in certain measurements, including tricuspid valve annular motion and inferior vena cava (IVC) diameter, was noted. For IVC assessment, the maximum and minimum diameters during quiet respiration were measured in the subcostal long-axis view using M-mode or 2D imaging. The absence of IVC respiratory variability was defined as <50% decrease in diameter during inspiration compared with that during expiration, in accordance with ASE guidelines.

A threshold of >21 mm or ≤21 mm was applied for tricuspid valve annular motion. The impact of reduced respiratory variation on hemodynamic status was also considered. Cardiac function was assessed based on echocardiographic parameters obtained using an ultrasound imaging system. Ultrasonographic examinations were performed using the GE Healthcare LOGIQ Series (GE Healthcare, Chicago, IL, USA), including the LOGIQ B and LOGIQ E95 models, as well as the Toshiba Aplio Series (Toshiba Medical Systems, Tochigi, Japan). In patients after the Fontan procedure, it is anatomically difficult to obtain standard echocardiographic measurements because of the absence of a functional right ventricle, altered systemic venous pathways, and variable postoperative connections between the vena cava, hepatic veins, and pulmonary arteries, which limit the applicability of conventional chamber and valvular assessment. In contrast, patients with repaired TOF generally retain biventricular anatomy, allowing for more complete and standardized echocardiographic data acquisition; therefore, we were able to perform the subgroup analysis in this cohort.

### 2.4. Abdominal Computed Tomography (CT)

Imaging examinations for the training cohort were conducted using one of the following three CT scanners: the SOMATOM Definition Flash (Siemens, Munich, Germany), the Aquilion ONE, and the Aquilion 64 (both from Canon Medical Systems, Otawara, Tochigi, Japan).

### 2.5. Liver Biopsy

Percutaneous liver biopsy was performed in five patients using a 21-gauge needle (MAJIMA needle; Hakko Co., Ltd., Medical Device Division, Nagano, Japan) with an ultrasound device (Prosound SSD-α10; HITACHI Aloka, Tokyo, Japan).

### 2.6. Blood Tests

Blood samples were collected during the outpatient stable phase to monitor overall health and organ function. The measured parameters included platelet count, alkaline phosphatase, albumin, gamma-glutamyl transpeptidase (γ-GTP), aspartate transaminase (AST), B-type natriuretic peptide (BNP), and alanine aminotransferase (ALT).

Liver fibrosis was assessed using the APRI as follows:

$$\mathrm{APRI}=\left(\frac{\mathrm{AST}\;}{\mathrm{Upper}\;\mathrm{Limit}\;\mathrm{of}\;\mathrm{Normal}\;\mathrm{AST}\;}\right)\times 100÷\mathrm{Platelet}\;\mathrm{Count}$$

AST was measured in IU/L, while platelet count was expressed in ×10^9^/L. Counts were measured in the peripheral blood samples collected during routine clinical evaluations. The upper limit of normal for AST was defined as 30 U/L based on our laboratory’s reference range.

The APRI was interpreted according to previously established thresholds, with values above >0.5 indicating suspected early-stage fibrosis. This method has been validated as a non-invasive tool for estimating liver fibrosis, particularly in pediatric patients [21].

As Fontan anatomy limited comprehensive echocardiographic evaluation and introduced missing data, we focused our subgroup analysis on patients after TOF repair, whose postoperative anatomy permits standardized assessment. We selected individuals aged ≥15 years—an age beyond which myocardial proliferation is largely complete [22]—to minimize size-related bias.

### 2.7. Statistical Analysis and Data Visualization

For the comparative analysis of echocardiographic parameters and liver fibrosis markers among the three groups (postoperative VSD cases, postoperative TOF cases, and postoperative Fontan procedure cases), statistical tests and graphical representations were performed as follows.

#### 2.7.1. Data Presentation and Analysis

Continuous variables are presented as medians and interquartile ranges due to their non-parametric distribution, while categorical variables are presented as counts (percentages). Group differences were assessed using the chi-square test for categorical variables and the Mann–Whitney U test for continuous variables. To compare three groups, Dunnett’s T3 test—which does not assume homogeneity of variances—was used. The Kruskal–Wallis test was applied to assess differences among groups due to non-normal data distribution. Statistical significance was set at *p* < 0.05.

#### 2.7.2. Visualization

To establish clinically actionable cutoff thresholds for routine biochemical assays at the point of care, we established a rapid, highly sensitive marker that can be measured instantly from routine outpatient blood draws. Receiver operating characteristic (ROC) curve analyses for BNP and γ-GTP were performed to determine optimal thresholds for identifying patients with APRI > 0.5, and to provide clinically applicable cutoff values that could be readily adopted across different patient populations and clinical settings. The area under the curve (AUC) was computed to evaluate the overall discriminative ability of BNP and γ-GTP.

Optimal thresholds for BNP and γ-GTP were identified by maximizing Youden’s J (sensitivity + specificity − 1) on the ROC curves. Additionally, we have reported the corresponding sensitivity and specificity at these thresholds.

#### 2.7.3. Statistical Tools

All statistical analyses were performed using SPSS Statistics version 27 (IBM, Tokyo, Japan) and Python (version 3.11.8; Python Software Foundation, Wilmington, DE, USA) with the scikit-learn library for ROC analysis. The optimal threshold was identified by minimizing the absolute difference between sensitivity and specificity.

### 2.8. Ethics Declarations

The study protocol was approved by the Ethics Committee of Niigata University on 26 August 2020 (approval number: 2020-0199) and conforms to the provisions of the Declaration of Helsinki. Written informed consent was not obtained because an opt-out procedure was used; participants were provided with study information and given the opportunity to decline participation.

## 3. Results

### 3.1. Patient Demographics and Group Characteristics

Baseline characteristics of the 142 patients are summarized in Table 1. The cohort was divided into VSD (n = 47), TOF (n = 67), and Fontan (n = 28) groups. Overall, 52.8% patients were male, with no significant difference in sex distribution among the groups. Median age was 22.7 years (range 0–77) and did not differ between the VSD (24.3 years) and TOF (24.0 years) groups but was significantly lower in the Fontan group (12.5 years) than in TOF (*p* < 0.05). CTR was significantly smaller in VSD (median 46.1%, range 41.0–50.0) than in TOF (54.2%, 44.0–68.0; *p* < 0.001), with no other intergroup differences.

### 3.2. Serum Biomarkers

Median brain natriuretic peptide (BNP) rose progressively from 11.2 pg/mL in VSD to 17.3 pg/mL in TOF and 27.8 pg/mL in Fontan (TOF vs. VSD, *p* < 0.01; Fontan vs. VSD and Fontan vs. TOF, both *p* < 0.001).

**Figure 2:**
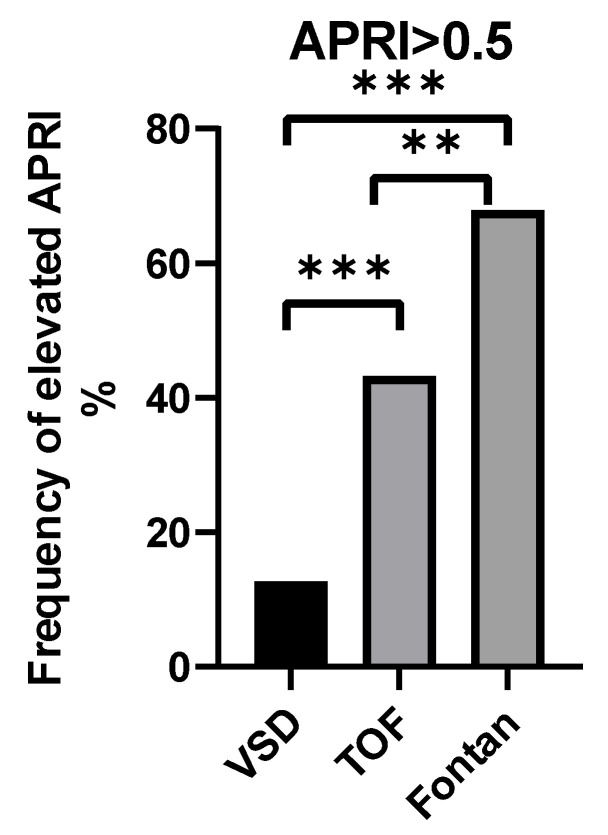
The frequency of APRI elevation (>0.5) in each CHD subgroup. ** *p* < 0.01, *** *p* < 0.001. APRI, aspartate aminotransferase-to-platelet ratio index; CHD. congenital heart disease.

The APRI score showed no difference between VSD (0.36) and TOF (0.40) but was higher in Fontan (0.55) than in VSD (*p* < 0.01) and TOF (*p* < 0.05). Likewise, the proportion of patients with an APRI > 0.5 increased from 12.7% in VSD to 43.3% in TOF and 67.9% in Fontan (VSD vs. TOF and VSD vs. Fontan *p* < 0.001; TOF vs. Fontan *p* < 0.01) (Figure 2).

**Figure 3:**
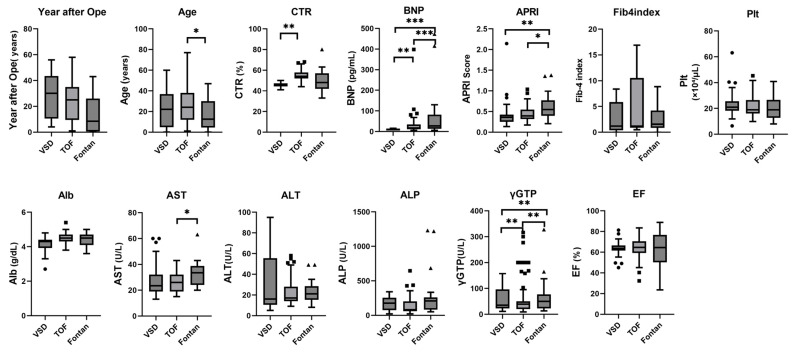
Box plot of data for patients with VSD, TOF, and post-Fontan surgery. * *p* < 0.05, ** *p* < 0.01, *** *p* < 0.001. BNP, B-type natriuretic peptide; γ-GTP, gamma-glutamyl transferase; Fib4, Fibrosis-4; AST, aspartate transaminase; ALT alanine aminotransferase; ALP, alkaline phosphatase; Alb, albumin; Plt, platelet; CTR, cardiothoracic ratio; EF, ejection fraction.

Liver enzymes showed selective elevations in the Fontan group: AST was higher in Fontan (33.5 U/L) than in TOF (26.0 U/L; *p* < 0.01), while γ-GTP increased stepwise across VSD, TOF, and Fontan (all comparisons *p* < 0.01). No significant differences were observed in platelet count, albumin, ALT, or ALP. Years since operation, ejection fraction, and the low prevalence of hepatocellular carcinoma (2.0% in Fontan) did not differ among the groups (Figure 3).

### 3.3. Echocardiographic Parameters

Absence of IVC respiratory variability was detected in 13.3% of patients overall (18/135) but was far more common in Fontan (39.3%) than in VSD (5.0%) or TOF (7.5%; all *p* < 0.01).

### 3.4. APRI Stratification and Early Fibrosis Risk

When stratified by fibrosis risk (Table 2), patients with an APRI > 0.5 (n = 54) had a markedly different group distribution than those with an APRI ≤ 0.5 (n = 88; VSD/TOF/Fontan: 41/38/9 vs. 6/29/19; *p* < 0.001). Although sex and age did not differ, those with APRI > 0.5 exhibited higher brain natriuretic peptide levels (median 19.8 vs. 13.8 pg/mL; *p* = 0.017), lower platelet counts (15.95 × 10^4^/µL vs. 23.2; *p* < 0.001), and elevated transaminases (AST: 32 vs. 22 U/L; ALT: 23.5 vs. 15.5 U/L; both *p* < 0.001) and γ-GTP (50 vs. 18 U/L; *p* < 0.001). Fibrosis-4 index was also higher in the APRI > 0.5 group (0.608 vs. 0.3255; *p* < 0.001), and absence of IVC variability was more common (29.6% vs. 2.3%; *p* < 0.05).

### 3.5. ROC Curve Analysis

**Figure 4:**
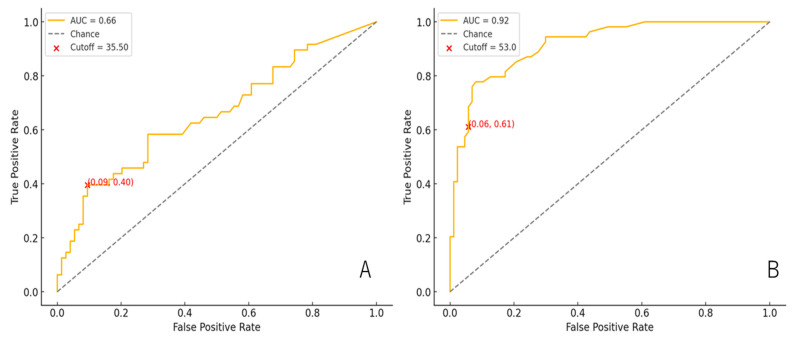
ROC curve of (**A**) BNP cutoff values in cases of fibrosis progression and (**B**) γ-GTP cutoff values in cases of fibrosis progression. ROC, receiver operating characteristic; AUC, area under the curve.

Based on the above results, receiver operating characteristic (ROC) analysis identified an optimal BNP and γ-GTP. BNP and γ-GTP, both obtainable from routine same-day blood draws, can serve as simple and sensitive markers for fibrosis screening. ROC curve analysis revealed an AUC of 0.66 for BNP, with an optimal cutoff of 35.5 pg/mL (sensitivity 39.6%; specificity 90.5%) (Figure 4A). For γ-GTP, the AUC was 0.94, with a threshold of 53.0 U/L yielding 61.1% sensitivity and 94.3% specificity (Figure 4B), indicating strong discriminative power for fibrosis risk assessment.

### 3.6. Analysis Limited to the TOF Population

Given that Fontan patients have underlying single-ventricle physiology and complex postoperative anatomical alterations that often preclude standard echocardiographic measurements, it is therefore more appropriate to compare echo parameters in postoperative TOF patients—whose preserved biventricular anatomy permits reliable assessment—once both groups have reached a predefined age threshold. We focused on individuals aged ≥15 years after TOF repair, whose postoperative anatomy allows standardized echocardiographic assessment (Table 3), minimizing size-related bias from ongoing myocardial proliferation. Baseline clinical and echocardiographic characteristics of the 49 patients with measurable data are summarized in Table 1. Twenty-four patients had an APRI  <  0.5 and 25 had an APRI  >  0.5. There were no significant differences in sex distribution (58.3% vs. 60.0% male; *p* = 0.264), median age (26.5 vs. 34.5 years; *p* = 0.059), or BNP levels (16.2 vs. 18.7 pg/mL; *p* = 0.942) between the two groups.

Patients with an APRI score greater than 0.5 exhibited a significantly higher cardiothoracic ratio compared to those with APRI ≤ 0.5 (median 55% vs. 52.5%; *p* = 0.019), along with notably lower platelet counts (16.1 × 10^4^/µL vs. 18.2 × 10^4^/µL; *p* = 0.002). Hepatic enzyme levels were markedly elevated in the APRI > 0.5 group, with AST (28.5 vs. 18 U/L; *p* < 0.001), ALT (27.5 vs. 16.5 U/L; *p* < 0.001), ALP (77 vs. 59.85 U/L; *p* = 0.002), and γ-GTP (45 vs. 23 U/L; *p* = 0.015) all demonstrating significant differences. Both the Fib-4 index (1.1685 vs. 0.6135; *p* = 0.001) and APRI score itself (0.589 vs. 0.3255; *p* < 0.001) were significantly higher in the APRI > 0.5 group.

Echocardiographic assessment revealed that patients with an APRI > 0.5 had greater posterior wall thickness during diastole (8.9 vs. 8.4 mm; *p* < 0.05) and increased left ventricular mass index (92 vs. 71.3 g/m^2^; *p* < 0.05). Furthermore, tricuspid regurgitation (TR) was more frequently observed in this group (16/23 [69.6%] vs. 8/23 [34.8%]; *p* < 0.01). No significant differences were found between the two groups in other echocardiographic parameters, including E/A ratio, e′ velocity, ventricular dimensions, septal thickness, TAPSE, chamber sizes, or the presence of valvular stenosis or regurgitation (excluding TR), right ventricular systolic pressure, or TR pressure gradient. Notably, absence of respiratory variation in the inferior vena cava (IVC) was observed exclusively in the APRI > 0.5 group (5/25 [20.0%] vs. 0/24 [0%]; *p* < 0.01).

### 3.7. Representative Patient Exhibiting Typical Clinical Features Observed in the Study Cohort

**Figure 5:**
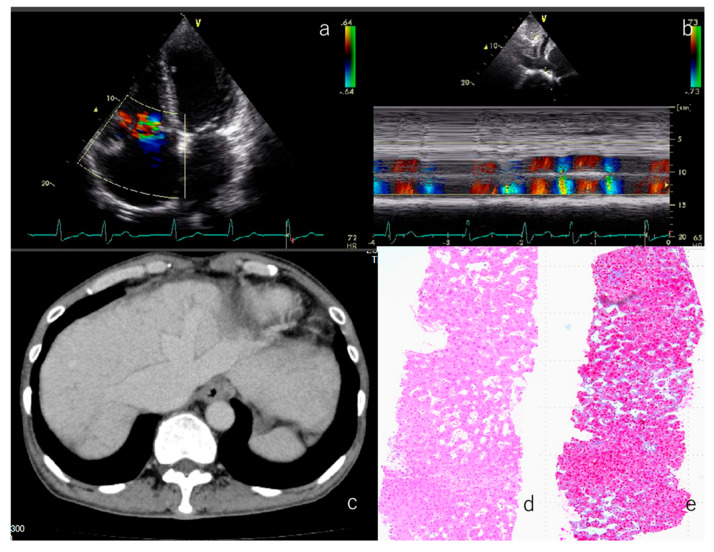
Representative patient exhibiting typical clinical features observed in the study cohort. (**a**) TR as a significant factor of fibrosis in the TOF group. (**b**) Absence of IVC respiratory variability, indicating hemodynamic impairment. (**c**) CT of the same patient demonstrating congestive hepatopathy with heterogeneous enhancement. (**d**,**e**) Histology findings: H&E staining (**d**); azan staining showing dilated hepatic veins and sinusoidal fibrosis (**e**). TR, tricuspid regurgitation; CT, computed tomography; H&E, hematoxylin and eosin.

Although liver biopsies were performed in five patients, these cases were not included in the main analysis due to the small sample size. Representative echocardiographic and imaging findings from a male patient 50 years after surgical repair of tetralogy of Fallot (TOF) demonstrate these typical features. Color Doppler imaging revealed tricuspid regurgitation in the apical four-chamber view (Figure 5a). Corresponding subcostal IVC views indicated an absence of respiratory variation, with minimal IVC collapse during respiration (Figure 5b). Abdominal CT imaging showed congestive hepatopathy characterized by heterogeneous hepatic enhancement, dilated hepatic veins, and periportal edema (Figure 5c). A liver biopsy specimen from the same patient confirmed histopathological features of congestive hepatopathy, including sinusoidal dilatation, centrilobular congestion, and hepatocyte atrophy (Figure 5d).

## 4. Discussion

The postoperative hepatic burden in CHD with right-sided overload is multifactorial and involves hepatic congestion, hypoxia-induced ischemia, a pro-thrombotic milieu, lymphatic congestion, and systemic inflammation [8]. Several reports have also highlighted Fontan-associated liver disease (FALD) as a clinical precursor of hepatocellular carcinoma (HCC): for example, a MELD-XI analysis of 154 postoperative Fontan patients [23] and a study emphasizing serial α-fetoprotein monitoring for HCC surveillance [24]. In a single-center cohort of 313 Fontan patients, cirrhosis rates increased from 1.3% at 5 years to 97.9% by 30 years post procedure [25], and a broader multi-institutional meta-analysis estimated that about 25% of adult Fontan survivors will develop cirrhosis in their lifetime [26].

In the present single-center, consistent-protocol design, we directly compared three cohorts—VSD, TOF, and post-Fontan—to identify early indicators of mild liver fibrosis. Our key findings were as follows:

Step-wise APRI elevation:

APRI increased in parallel with right-sided hemodynamic load, from a median of 0.36 in VSD (12.7% with APRI > 0.5) to 0.40 in TOF (43.3%) and 0.55 in Fontan (67.9%) (VSD vs. Fontan *p* < 0.001; TOF vs. Fontan *p* < 0.01). This clear, graded escalation underscores the direct impact of increasing volume and pressure overload on hepatic fibrosis.

High-specificity blood markers:

Two routinely available blood tests—BNP ≥ 35.5 pg/mL (specificity 90.5%) and γ-GTP ≥ 53 U/L (specificity 94.3%)—each accurately identified APRI > 0.5, offering a simple “same-day” screening strategy that reflects both cardiac and hepatic stress (Figure 4).

Echocardiographic correlation:

Absence of respiratory variability in the IVC was seen in 39.3% of Fontan patients but only 5.0% of VSD and 7.5% of TOF patients (*p* < 0.01), confirming its value as a quantitative measure of chronic right-sided congestion.

TOF-specific myocardial–hepatic links:

In a subgroup of TOF patients ≥ 15 years old (preserved biventricular anatomy), those with an APRI > 0.5 exhibited higher rates of tricuspid regurgitation (69.6% vs. 34.8%; *p* < 0.01) and increased left-ventricular posterior wall thickness and mass index (both *p* < 0.05), suggesting that subclinical myocardial remodeling accompanies early fibrosis.

Taken together, APRI, BNP, γ-GTP, and IVC respiratory variability form an inexpensive, bedside “triad” for detecting subclinical liver fibrosis in congenital heart disease survivors. Given the pronounced progression of fibrosis in Fontan patients, routine application of these three indicators could enable earlier intervention and potentially improve long-term hepatic outcomes. While the APRI was used as a reference marker in our analyses, the present study was not intended to validate its diagnostic performance for hepatic fibrosis. Several factors should be considered when interpreting our findings: the study cohort included pediatric patients for whom age-dependent formulas may be inappropriate; liver biopsy, the gold standard, was not feasible due to its invasive nature; the retrospective design limited the availability of uniform clinical data; and emerging fibrosis markers such as M2BPGi or autotaxin were not systematically collected. In the absence of direct comparison with gold-standard methods, our results should, therefore, be regarded as hypothesis-generating, serving to guide the design of future prospective validation studies. In the TOF subgroup, we noted a near-significant age difference between patients with and without advanced hepatic fibrosis. Although not statistically significant, age is a recognized factor influencing hepatic and cardiac remodeling, warranting cautious interpretation. Over time, even modest hepatic loading detectable on periodic echocardiography may contribute to fibrosis progression, underscoring the need for larger, longitudinal studies to clarify the confounding role of age and cumulative subclinical hepatic stress.

TOF remains one of the most common cyanotic CHDs—3–5% of all CHDs, ~0.28 per 1000 live births without sex predilection [27]—and was first described by Étienne-Louis Fallot in 1888 [28,29]. While most TOF cases are sporadic, ~6% harbor 22q11.2 microdeletions. The characteristic lesions—pulmonary stenosis, right-ventricular hypertrophy, VSD, and overriding aorta—arise from anterior deviation of the infundibular septum, elevating right-ventricular pressure (often ~100 mmHg vs. normal ~30 mmHg) [30] and causing cyanosis. Standard management is early total repair within the first year of life; advances in catheter-based techniques and prosthetic valves have led to ~85% long-term adult survival [31]. However, late complications—pulmonary-valve regurgitation (linked to oversized trans-annular patches or commissurotomy injury [32]), right-ventricular dysfunction, atrial arrhythmias, and infective endocarditis—predispose patients to both heart failure and hepatic dysfunction.

Retrospective studies have demonstrated elevated fibrosis markers (type IV collagen, hyaluronic acid) in TOF [32], and chronic hypoxia with elevated central venous pressure has been histologically associated with liver fibrosis in HCC and cholangiocarcinoma [33]. In patients with APRI > 0.5, raised CVP led to loss of IVC respiratory variability, increased right-heart volume load, and aggravated tricuspid regurgitation, alongside compensatory left-ventricular hypertrophy (posterior wall thickening, increased LV mass index)—all occurring despite preserved systolic and key diastolic function, indicating that remodeling precedes overt dysfunction.

Accordingly, non-invasive fibrosis assessment in TOF has gained traction, with serum indices such as type IV collagen, hyaluronic acid, APRI, and FIB-4 widely applied [34]. The APRI itself—a ratio of AST to platelet count proposed by Wai et al. in 2003 [35]—remains a simple fibrosis marker endorsed by WHO hepatitis B guidelines (APRI > 0.5 or elastography > 7.0 kPa for significant fibrosis; APRI > 1.0 or >12.5 kPa for cirrhosis) [36]. While FIB-4 (age × AST ÷ [platelets × √ALT]) [37] performs well in adults, it lacks pediatric applicability due to its age component [38]. In Egyptian children with chronic hepatitis C, APRI outperformed FIB-4 for significant fibrosis [39], and in chronic hepatitis B, APRI ≥ 0.5 and FIB-4 showed comparable AUCs (0.864) for advanced fibrosis, with APRI excluding 95.4% of F3/F4 cases when elastography is unavailable [40,41].

Elastography (ultrasound or MRI) quantifies liver stiffness but may overestimate fibrosis in both CHD and non-CHD heart failure and is not yet recommended for routine CHD follow-up [42,43]. By focusing on APRI ≥ 0.5, our identification of γ-GTP [44] and BNP as practical surrogates simplifies transitional care from pediatric cardiology to adult hepatology. We plan to validate this triad in larger prospective cohorts. In the TOF subgroup, increased left ventricular wall thickness and the presence of tricuspid regurgitation may contribute to hepatic fibrosis through mechanisms involving right ventricular dysfunction and elevated systemic venous pressure, which can exacerbate chronic hepatic congestion.

This study has some limitations. This retrospective study lacked systematic data on emerging fibrosis markers (e.g., Mac-2 binding protein glycosylation isomer and autotaxin), with pediatric reference ranges remaining undefined. Detailed stratification by TOF subtype was limited; moreover, prosthetic valves can mask echocardiographic abnormalities. Furthermore, we aimed to furnish hepatologists and general internists with an initial risk assessment tool rather than a fully validated algorithm. Future large-scale prospective studies incorporating spleen size, additional biomarkers, and standardized elastography are warranted.

One of the major strengths of this study is its single-center design, which ensured uniformity in surgical procedures, follow-up intervals, and ultrasonographic techniques. This methodological consistency enhances the reliability of intergroup comparisons. Moreover, the inclusion of patients with repaired VSD as a control group allowed for accurate evaluation of disease-specific differences in hepatic outcomes among various congenital heart disease subtypes.

The single-center design ensured consistent procedures and imaging protocols but limits the generalizability of our findings to other settings. In addition, the small sample sizes, particularly in the Fontan and TOF sub-analyses, reduce statistical power; therefore, negative results should be interpreted with caution.

## 5. Conclusions

Liver fibrosis is a long-term complication in patients with congenital heart disease, especially those who have undergone Fontan surgery or TOF repair. The use of non-invasive markers—particularly APRI, γ-GTP, and BNP—in combination with echocardiographic parameters is a feasible strategy for early identification of hepatic involvement. Given their availability in routine clinical settings, these markers may support timely referrals to hepatology and facilitate integrated follow-up across pediatric and adult care. Establishing standardized screening protocols could improve long-term outcomes and help bridge the gap between cardiac and hepatic care in this growing patient population.

## Figures and Tables

**Table 1:** Clinical and laboratory characteristics of patients by postoperative cardiac condition.

Parameter	Total (n = 142)	Postoperative VSD (n = 47)	Postoperative TOF (n = 67)	Postoperative Fontan (n = 28)	VSD vs. TOF	VSD vs. Fontan	TOF vs. Fontan
Male (%)	52.8% (75/142)	46.8% (22/47)	56.7% (38/67)	53.5% (15/28)	n.s.	n.s.	n.s.
Year after operation (years)	28.0 (1–58)	37.0 (25–56)	25.0 (1–58)	10.0 (1–40)	n.s.	n.s.	n.s.
Age (years)	22.7 (0–77)	24.3 (0–60)	24.0 (1–77)	12.5 (0–47)	n.s.	n.s.	*
Cardiothoracic Ratio (%)	50.8 (33–80)	46.1 (41–50)	54.2 (44–68)	48.0 (33–80)	***	n.s.	n.s.
BNP (pg/mL)	15.0 (5.8–472.8)	11.2 (5.8–16.1)	17.3 (5.8–398.0)	27.8 (5.8–472.8)	**	***	***
APRI Score	0.39 (0.13–2.14)	0.36 (0.13–2.14)	0.40 (0.17–1.31)	0.55 (0.21–1.38)	n.s.	**	*
APRI > 0.5 (%)	38.1% (54/142)	12.7% (6/47)	43.3% (29/67)	67.9% (19/28)	***	***	**
Fib4 Index	0.7 (0.0–16.9)	1.20 (0.0–8.39)	1.21 (0.05–16.9)	1.56 (0.00–8.84)	n.s.	n.s.	n.s.
Platelet count (×10^4^/μL)	20.0 (6.5–63.1)	21.0 (6.5–63.1)	18.9 (9.9–45.3)	19.0 (8.0–40.8)	n.s.	n.s.	n.s.
Albumin (g/dL)	4.5 (2.7–5.4)	4.3 (2.7–4.8)	4.5 (3.8–5.4)	4.5 (3.6–5.0)	n.s.	n.s.	n.s.
AST (U/L)	26.3 (13–63)	23.5 (13–60)	26.0 (15–43)	33.5 (20–63)	n.s.	n.s.	**
ALT (U/L)	18.0 (5–95)	16.0 (5–95)	17.0 (9–58)	21.0 (8–49)	n.s.	n.s.	n.s.
ALP (U/L)	105.4 (21.4–1229.7)	67.0 (22–340)	80.0 (21.4–645)	209.8 (51.1–1229.7)	n.s.	n.s.	n.s.
γ-GTP (U/L)	26.0 (9–327)	35.0 (11–157)	42.0 (9–316)	50.0 (13–327)	**	**	**
Ejection Fraction (%)	64.2 (23.6–88.9)	63.3 (45.1–70.0)	64.7 (32.3–83.5)	64.5 (23.6–88.9)	n.s.	n.s.	n.s.
Absence of IVC respiratory variability (%)	13.3% (18/135)	5.0% (2/40)	7.5% (5/67)	39.3% (11/28)	**	**	**
Hepatocellular Carcinoma (%)	2.0% (2/142)	0% (0/47)	0% (0/67)	7.1% (2/28)	n.s.	n.s.	n.s.

* *p* < 0.05, ** *p* < 0.01, *** *p* < 0.001. IVC, inferior vena cava; n.s., not significant; VSD, ventricular septal defect; TOF, tetralogy of Fallot; BNP, B-type natriuretic peptide; γ-GTP, gamma-glutamyl transferase; APRI, aspartate aminotransferase-to-platelet ratio index; Fib4, Fibrosis-4; AST, aspartate transaminase; ALT alanine aminotransferase; ALP, alkaline phosphatase.

**Table 2:** Comparison of clinical characteristics by APRI category.

Parameters	APRI < 0.5 (n = 88)	APRI > 0.5 (n = 54)	*p*-Value
Postoperative (VSD/TOF/Fontan)	41/38/9	6/29/19	<0.001
Male (%)	48.9% (43/88)	57.4% (31/54)	0.372
Years after operation (years)	28.0 (1–58)	37.0 (25–56)	0.749
Age (years)	21.2 (0–67)	27.0 (0–77)	0.117
Cardiothoracic Ratio (%)	49.9 (33–63)	46.1 (41–80)	0.542
BNP (pg/mL)	13.8 (5.8–129.7)	19.8 (5.8–472.8)	0.017
APRI score	0.33 (0.13–0.48)	0.61 (0.43–2.14)	<0.001
Fib4 Index	0.33 (0.13–0.48)	0.61 (0.43–2.14)	<0.001
Platelet count (×10^4^/μL)	23.2 (13.3–63.1)	16.0 (6.5–27.0)	<0.001
Albumin (g/dL)	4.5 (2.7–5.0)	4.5 (3.6–5.4)	0.321
AST (U/L)	22.0 (13–57)	32.0 (19–63)	<0.001
ALT (U/L)	15.5 (5–95)	23.5 (10–75)	<0.001
ALP (U/L)	81.6 (40–1229.7)	114.1 (21.4–1218.4)	0.797
γ-GTP (U/L)	18.0 (9–129)	50.0 (12–327)	<0.001
Ejection Fraction (%)	64.2 (23.6–88.9)	63.3 (45.1–70.0)	0.335
Absence of IVC respiratory variability (%)	2.3% (2/88)	29.6% (16/54)	<0.05

**Table 3:** Baseline characteristics and echocardiographic parameters by APRI categories.

Parameters	Total (n = 49)	APRI < 0.5 (n = 24)	APRI > 0.5 (n = 25)	*p*-Value
Sex, Male (%)	59% (29/49)	58.3% (14/24)	60% (15/25)	0.264
Years after operation (years)	30.5 (3–58)	28.5 (19–58)	31 (3–56)	0.545
Age (years)	31 (15–77)	26.5 (15–67)	34.5 (16–77)	0.059
Cardiothoracic Ratio (%)	54 (44–68)	52.5 (44–61)	55 (48–68)	0.019
BNP (pg/mL)	16.6 (5.8–398)	16.2 (5.8–81.2)	18.65 (5.8–398)	0.942
APRI Score	0.4195 (0.17–1.04)	0.3255 (0.17–0.41)	0.589 (0.43–1.04)	<0.001
Fib4 Index	0.8005 (0.25–5.23)	0.6135 (0.25–1.82)	1.1685 (0.42–5.23)	<0.001
Platelet Count (×10^4^/μL)	17.85 (9.9–38.6)	18.2 (15.2–38.6)	16.1 (9.9–23.1)	<0.01
Albumin (g/dL)	4.5 (3.8–5.4)	4.5 (4.0–4.9)	4.5 (3.8–5.4)	0.983
AST (U/L)	23 (15–42)	18 (15–39)	28.5 (19–42)	<0.001
ALT (U/L)	21 (9–58)	16.5 (9–37)	27.5 (14–58)	<0.001
ALP (U/L)	63.9 (21.4–286)	59.9 (40–134.4)	77 (21.4–286)	0.002
γ-GTP (U/L)	27 (11–316)	23 (11–129)	45 (12–316)	0.015
Ejection Fraction (%)	64.1 (40.4–81.4)	63.9 (40.4–81.4)	64.2 (45.1–80.3)	0.550
E/A Ratio	1.55 (0.6–3.14)	1.55 (0.79–3.14)	1.49 (0.6–2.69)	0.408
e′ velocity (cm/s)	8.05 (3.9–16.9)	8.05 (3.9–15)	8.1 (4.4–16.9)	0.488
LV Internal Diameter diastole (mm)	45.1 (37.5–64.6)	43.8 (38.3–64.6)	46.15 (37.5–56)	0.139
LV Internal Diameter systole (mm)	30.4 (21.8–45.5)	29.6 (21.8–45.5)	30.6 (22.8–37.9)	0.060
Interventricular Septal Thickness (mm)	8.7 (4.2–13.9)	8.7 (5.6–10.4)	9.05 (4.2–13.9)	0.274
Posterior Wall Thickness in diastole (mm)	8.6 (3.8–11.2)	8.4 (3.8–10.7)	8.9 (5.2–11.2)	<0.05
Left Ventricular Mass Index (g/m^2^)	78.6 (52.1–170)	71.3 (52.1–151.9)	92 (62.1–170)	<0.05
TAPSE (mm)	16.7 (1.8–28.5)	16.8 (1.8–28.5)	16.65 (7.8–25)	0.991
Right Ventricular Diameter (mm)	41.3 (19.1–58.1)	39.5 (19.1–58.1)	43.2 (19.2–53.2)	0.419
Left Atrial Diameter (mm)	36.2 (23.4–50.4)	32.55 (23.4–45.4)	36.6 (24.2–50.4)	0.716
Left Atrial Volume Index (mL/m^2^)	23.3 (12.1–86.6)	19.8 (12.1–41.2)	25.8 (12.8–86.6)	0.477
Right Atrial Diameter (mm)	29.8 (5–61)	29.8 (14.9–54)	31.45 (5–61)	0.942
Tricuspid Regurgitation (%)	24/46 (52.2%)	8/23 (34.8%)	16/23 (69.6%)	<0.01
Pulmonary Regurgitation (%)	21/47 (44.7%)	10/26 (38.5%)	11/21 (52.4%)	0.630
Tricuspid Regurgitation Pressure Gradient (mmHg)	26 (14.4–74)	27 (15.7–74)	25 (14.4–65.5)	0.903
Inferior Vena Cava Diameter (mm)	15 (8.7–24)	14.75 (8.7–17.5)	15.3 (10.4–24)	0.341
Absence of IVC respiratory variability (%)	5/25 (20.0%)	0/24 (0%)	5/25 (20.0%)	<0.01

E/A ratio, ratio of early to late mitral inflow velocities; TAPSE, tricuspid annular plane systolic excursion; LV, left ventricle.

## Data Availability

The data presented in this study are available on request from thecorresponding author. The data are not publicly available due to the restrictions eg privacy or ethical.

## Abbreviations

The following abbreviations are used in this manuscript:

APRI	Aspartate aminotransferase-to-platelet ratio index
CHD	Congenital heart disease
IVC	Inferior vena cava
n.s.	Not significant
VSD	Ventricular septal defect
TOF	Tetralogy of Fallot
BNP	B-type natriuretic peptide
γ-GTP	Gamma-glutamyl transferase
Fib-4	Fibrosis-4
AST	Aspartate transaminase
ALT	Alanine aminotransferase
ALP	Alkaline phosphatase
Alb	Albumin
Plt	Platelet
RA	Right atrium
RV	Right ventricle
LA	Left atrium
LV	Left ventricle
PA	Pulmonary artery
AO	Aorta
CTR	Cardiothoracic ratio
EF	Ejection fraction
ROC	Receiver operating characteristic
AUC	Area under the curve
E/A ratio	Ratio of early to late mitral inflow velocities
TAPSE	Tricuspid annular plane systolic excursion
TR	Tricuspid regurgitation
CT	Computed tomography
H&E	Hematoxylin and eosin
